# Outer membrane vesicles from *Akkermansia muciniphila* antagonize chronic stress-induced colorectal cancer progression by downregulating Fetuin-A

**DOI:** 10.3389/fmicb.2026.1821362

**Published:** 2026-05-22

**Authors:** Xinyu Zhang, Yanjie Lu, Shunkang Jin, Yanzhen Zuo, Hongyan Zuo, Yanhui Hao, Qian Xu, Mengyun Wang, Yang Li, Yuhong Li

**Affiliations:** 1 Cancer Research Laboratory, Chengde Medical College, Chengde, China; 2 Institute of Radiation Medicine, Academy of Military Medical Sciences, Beijing, China

**Keywords:** *Akkermansia muciniphila*, chronic stress, colorectal cancer, Fetuin-A, outer membrane vesicles

## Abstract

**Introduction:**

Chronic stress is known to exacerbate the malignant progression of colorectal cancer (CRC), a leading cause of cancer-related mortality worldwide. While outer membrane vesicles (OMVs) derived from *Akkermansia muciniphila* (*A. muciniphila*) exhibit anti-tumor potential, their underlying mechanisms in the context of stress remain unclear. This study investigated whether *A. muciniphila* OMVs could mitigate chronic stress-induced CRC progression by regulating the pro-tumorigenic protein Fetuin-A.

**Methods and results:**

*In vitro*, the *β*-adrenergic agonist isoproterenol (ISO), used to simulate chronic stress, significantly promoted CRC cell proliferation, migration, and invasion while inhibiting cellular uptake of OMVs. Notably, *A. muciniphila* OMVs effectively rescued the ISO-driven malignant progression in CRC cells by counteracting the upregulation of Fetuin-A. *In vivo*, using a CRC mouse model combined with chronic unpredictable mild stress, we found that OMV administration markedly suppressed tumor growth. This therapeutic effect was associated with a significant downregulation of Fetuin-A expression in tumor tissues.

**Conclusion:**

Collectively, our findings demonstrate that *A. muciniphila* OMVs inhibit chronic stress-driven CRC progression by downregulating Fetuin-A. This study uncovers a novel regulatory mechanism within the “stress–Fetuin-A–CRC” axis and highlights *A. muciniphila* OMVs as a promising therapeutic strategy for managing stress-associated CRC.

## 1 Introduction

Colorectal cancer (CRC) poses a significant global public health challenge, consistently ranking among the leading malignancies in terms of both incidence and mortality (Chen Y. et al., 2024; Morgan et al., 2023). The pathophysiology of CRC is highly complex, with its progression intricately regulated by a confluence of factors, including genetic alterations (Huang et al., 2021; Wang et al., 2023; Ouyang et al., 2024), environmental exposures (Adamczyk et al., 2021), and host physiological state (Han et al., 2024). In recent years, oncological research has undergone a notable paradigm shift, moving from a singular focus on cell-autonomous abnormalities within tumor cells toward a deeper exploration of the intricate interplay between tumors and their hosts (Cui et al., 2025; Zhao et al., 2021). Against this backdrop, psychological stress has emerged as a critical host factor, increasingly recognized for its role in remodeling the tumor microenvironment and driving the malignant progression of CRC; however, its underlying molecular mechanisms remain incompletely understood (Cui et al., 2025).

Chronic stress, a state of sustained physiological dysregulation, is established as a key driver of CRC malignancy (McCollum and Shah, 2024; Guan et al., 2023; Cao et al., 2024). At its core, the mechanism involves the sustained hyperactivation of the sympathetic nervous system by chronic stress. This disrupts the homeostatic balance of the complex neuro-endocrine-immune network, leading to chronically elevated levels of circulating catecholamines such as norepinephrine (Guan et al., 2023; Provost et al., 2023). These neurotransmitters, acting as crucial molecular mediators of the stress response, bind to *β*-adrenergic receptors on CRC cells, thereby activating downstream canonical pro-oncogenic signaling cascades (Varghese et al., 2025), including the ERK1/2 and PI3K/AKT pathways (Zhi et al., 2023). Activation of these pathways endows tumor cells with a repertoire of malignant phenotypes, including accelerated proliferation and enhanced invasive and metastatic potential (McCollum and Shah, 2024), while further remodeling the tumor microenvironment to promote angiogenesis and induce immune evasion (Fernandes et al., 2022). Despite this established signaling axis from stress to tumor aggravation, the key downstream molecular effectors that execute these stress-induced commands and mediate phenotypic transformation in CRC cells remain to be fully identified.

Recent advances in microbiome research have highlighted the therapeutic potential of probiotics and their derivatives. Among these, *Akkermansia muciniphila* (*A. muciniphila*) has attracted considerable attention due to its excellent biocompatibility and promising anti-tumor properties (Jin et al., 2025). As a keystone species crucial for maintaining intestinal barrier integrity and metabolic homeostasis, *A. muciniphila has* been shown to effectively suppress CRC growth (Jin et al., 2025; Ghotaslou et al., 2023; Wade et al., 2023). Its secreted outer membrane vesicles (OMVs), which function as natural nanocarriers encapsulating diverse bioactive molecules, facilitate inter-kingdom communication between bacteria and host cells (Ghosh and Mani, 2022). Despite the established pro-tumorigenic role of chronic stress and the promising anti-CRC properties of *A. muciniphila* OMVs, it remains unknown whether these OMVs can counteract the specific aggravating effects of chronic stress on CRC progression. Moreover, the molecular mechanisms through which stress signaling and OMVs might interact are largely undefined. In particular, the identification of key effector proteins that mediate both stress-induced malignancy and the potential therapeutic action of OMVs represents an unexplored area of significant scientific and translational interest.

To address these gaps, the present study aimed to investigate whether *A. muciniphila* OMVs can mitigate chronic stress-driven CRC progression by regulating the expression of the pro-tumorigenic protein Fetuin-A. We hypothesized that chronic stress promotes CRC malignancy through upregulation of Fetuin-A, and that *A. muciniphila* OMVs exert their therapeutic effect by downregulating this critical protein. Using both *in vitro* models of adrenergic stimulation and *in vivo* models combining chronic unpredictable mild stress (CUMS) with colitis-associated CRC, we sought to: (1) characterize the impact of chronic stress on CRC cell phenotypes and OMVs uptake; (2) validate Fetuin-A as a stress-responsive mediator; and (3) evaluate the ability of *A. muciniphila* OMVs to reverse stress-induced tumor progression via Fetuin-A downregulation. By elucidating this “stress–Fetuin-A–CRC” regulatory axis and the modulatory role of bacterial OMVs, this work may provide novel mechanistic insights and identify a potential therapeutic strategy for managing stress-associated CRC.

## 2 Materials and methods

### 2.1 Animals

A total of 42 specific pathogen-free male C57BL/6 J mice (8 weeks old, 20–24 g body weight) were obtained from SPF (Beijing) Biotechnology Co., Ltd. (China). Mice were maintained under standard conditions (22 ± 2 °C, 55 ± 5% relative humidity, 12 h light/dark cycle) with ad libitum access to food and water. All animal experiments were approved by the Ethics Committee of the Experimental Animal Center of the Academy of Military Medical Sciences (Approval No.: IACUC-DWZX-2022–854) and conducted in accordance with the National Institutes of Health Guide for the Care and Use of Laboratory Animals.

### 2.2 Establishment of the azoxymethane (AOM)/dextran sodium sulfate (DSS)-induced colitis-associated CRC model

The colitis-associated CRC model was established using the classic AOM/DSS protocol (Liu et al., 2024). On day 0, all mice received a single intraperitoneal (i.p.) injection of AOM (10 mg/kg; Sigma-Aldrich, United States) dissolved in sterile normal saline. One week later, DSS (molecular weight 36–50 kDa; MP Biomedicals, United States) treatment was initiated. Mice underwent three cycles of 2.0% (w/v) DSS administered in drinking water for 7 consecutive days, each followed by a 14 day recovery period with regular sterile water. Subsequently, the mice were randomly assigned to either a control group or a stress group (*n* = 21 per group) using a computer-generated random sequence to ensure unbiased allocation. The mice in the stress group were then subjected to a CUMS protocol to establish the stress model.

### 2.3 Establishment of the CUMS model

Anxiety- and depression-like behaviors were induced using a 14 day CUMS protocol. Daily between 9:00 a.m. and 5:00 p.m., mice in the stress group were randomly subjected to one of the following stressors; the same stressor was not applied on consecutive days (Wang et al., 2022; Xu et al., 2023): (1) 24-h food deprivation (water freely available); (2) 24-h water deprivation (food freely available); (3) Cage tilt at 45° on wet bedding for 24 h (200 mL water added); (4) Restraint stress for 6 h in a ventilated 50 mL centrifuge tube; (5) Tail pinch stress for 30 min (hemostatic clamp applied 1 cm from the tail tip); (6) 24-h reversed light/dark cycle. Control mice were housed in the same room and handled identically, but were not exposed to stressors. Body weight, food intake, and water consumption were monitored, and disease activity index (DAI) was scored throughout the modeling period. The DAI system is based on three components: body weight loss ratio, stool consistency, and intestinal condition. The DAI was scored based on a previously published consensus report (Jin et al., 2025).

### 2.4 Behavioral assessment

At 24-h after CUMS model establishment, anxiety- and depression-like behaviors were assessed using the open-field test (OFT) and tail-suspension test (TST). To minimize stress-induced artifacts, mice were acclimated to the testing room for 30 min/day and handled by the experimenter for three consecutive days before behavioral testing. Tests were performed in a sound-attenuated room, with one test per day to prevent fatigue and a 60-min acclimation period before each session. Apparatuses were thoroughly cleaned with 75% ethanol between trials.

### 2.5 OPT

The OFT was conducted to evaluate anxiety-like behavior. Each mouse was individually placed in the center of a square arena (40 × 40 × 35 cm) facing away from the experimenter and allowed to explore freely for 5 min. Movements were tracked using an automated system (ANY-maze software, v6.32, Stoelting, United States). The arena was digitally divided into a central zone (20 × 20 cm) and a peripheral zone for analysis.

### 2.6 TST

The TST was used to assess depression-like behavior. Mice were suspended by the tail with medical tape inside a three-walled chamber (49 × 29 × 45 cm) for 6 min. Activity was recorded and analyzed with an automated video-tracking system (DigBehv, v4.0; Shanghai Jiliang Software Technology Co., Ltd., China). Immobility time was calculated from the final 4 min of the test.

### 2.7 Bacterial culture and OMVs preparation

*A. muciniphila* ATCC BAA-835 and *Escherichia coli* B98154 were purchased from Ningbo Minghai Biotechnology Co., Ltd. (China). The 16S rRNA gene sequence of the *A. muciniphila* strain is provided in Supplementary Data Sheet S1. The *E. coli* strain was used as a control. To ensure metabolic stability and consistent OMV secretion, *A. muciniphila* ATCC BAA-835 was revived from cryopreserved stocks (stored in 20% glycerol at −80 °C) and subcultured for only a single passage in Chopped Meat Carbohydrate (CMC) broth to minimize phenotypic drift (Cao et al., 2026; Kim et al., 2025). For OMV production, the bacteria were cultured in CMC broth at 37 °C for 72 h in an anaerobic chamber (Bactron EZ-2, Shellab, United States) with a controlled atmosphere of 80% N₂, 10% H₂, and 10% CO₂. The use of CMC broth was specifically chosen as it provides a superior low redox potential through its chopped meat components, acting as a natural reducing agent that mimics the anaerobic niches of the gut more effectively than standard synthetic media. This medium ensures robust growth of this obligate anaerobe while maintaining the integrity of the secreted vesicles. Bacterial cultures were then centrifuged at 8,000 rpm (approx. 7400×g ) for 10 min at 4 °C to remove cells. Bacterial cultures were centrifuged at 5000×g for 10 min at 4 °C to remove cells, and the supernatant was further centrifuged at 15000×g for 20 min at 4 °C, followed by filtration through a 0.22 μm filter. OMVs were pelleted by ultracentrifugation at 170000×g for 80 min at 4 °C, washed once in sterile PBS under the same centrifugation conditions, resuspended in sterile PBS, filter-sterilized (0.22 μm), and stored at −80 °C until use (Zhang et al., 2023). To verify the sterility of the purified OMVs, the samples were spread onto anaerobic agar plates and incubated at 37 °C for 72 h under anaerobic conditions; simultaneously, *A. muciniphila* was spread onto the same medium as a positive control. OMVs protein concentration was determined using the NanoPhotometer^®^ N60 (Implen, Munich, Germany). To characterize the morphology of the purified OMVs, samples were fixed and loaded onto carbon-coated copper grids, followed by negative staining with 2% (w/v) uranyl acetate. The ultrastructure and size distribution of OMVs were then visualized and photographed using a transmission electron microscopy (TEM). Concurrently, nanoparticle tracking analysis (NTA) was performed to accurately determine their size distribution and particle concentration. Furthermore, the protein composition of the OMVs was analyzed using SDS-PAGE. The samples were denatured and loaded onto a 10% (w/v) polyacrylamide gel. After electrophoresis, the gel was stained with Coomassie Brilliant Blue R-250 to determine the protein distribution and characterize the biochemical composition of the vesicles. In addition, Western blotting was performed to detect outer membrane proteins and the typical OMV marker LPS with anti-LPS antibody (Abmart, MK52671, 1:1000).

### 2.8 *In vivo* intervention

Mice were randomly assigned to five experimental groups (*n* = 7 per group) using a computer-generated random sequence to ensure unbiased allocation: Control, Stress, *A. muciniphila* OMVs, Stress + *A. muciniphila* OMVs, and Stress + *A. muciniphila* OMVs + Fetuin-A. During the 14-day intervention period, mice in the OMV-treated groups received daily oral gavage of 100 μL PBS containing 20 μg *A. muciniphila* OMVs (based on total protein) (Zhuang et al., 2021). For the Stress + *A. muciniphila* OMVs + Fetuin-A group, a single dose of recombinant mouse Fetuin-A (50 mg/kg; Meilunbio, Dalian, China) was administered via tail-vein injection on day 1 (Zhao P. et al., 2022). Except for the Stress + *A. muciniphila* OMVs + Fetuin-A group, anxiety- and depression-like behaviors in the remaining groups were assessed using the OFT and TST 24 h after the completion of the intervention. Finally, all mice were euthanized under pentobarbital anesthesia. The entire colorectum was excised, opened longitudinally, rinsed with ice-cold PBS, and examined for tumor number, size, and location.

### 2.9 Immunohistochemistry

Paraffin-embedded CRC tissue sections (4 μm) were deparaffinized, rehydrated, and subjected to antigen retrieval in citrate buffer (pH 6.0) at 95 °C for 20 min. Endogenous peroxidase activity was quenched with 3% H₂O₂ for 15 min. Sections were incubated overnight at 4 °C with anti-Fetuin-A antibody (1:20; Proteintech, 66,094-1-Ig, Wuhan, China), followed by incubation with a biotinylated secondary antibody for 30 min at 37 °C. Color was developed using DAB, and nuclei were counterstained with hematoxylin. Sections were dehydrated, cleared, mounted, and imaged under a light microscope.

### 2.10 Cell culture

Human CRC cell lines HCT-116 and HT-29 were obtained from ATCC (United States). HCT-116 cells were maintained in high-glucose Dulbecco’s Modified Eagle’s Medium (Gibco), and HT-29 cells in McCoy’s 5A Medium (Gibco). Both media were supplemented with 10% fetal bovine serum (FBS, Gibco) and 1% penicillin–streptomycin solution (Gibco). All cells were cultured in a humidified incubator at 37 °C under 5% CO₂ and routinely passaged upon reaching 80–90% confluence.

Standardized *in vitro* treatments were applied across functional assays unless otherwise specified. Cells were treated as follows: (1) Control group: fresh complete medium containing an equivalent volume of PBS; (2) Isoproterenol (ISO) group: medium supplemented with 20 μM ISO (Solarbio, II02002, Beijing, China) to simulate chronic adrenergic signaling (Liu Y. et al., 2022); (3) ISO + OMVs group: medium containing 20 μM ISO and 20 μg/mL of *A. muciniphila* OMVs (total protein). For the OMVs-only condition in specific experiments, cells were treated with 20 μg/mL OMVs in the absence of ISO. Treatments were initiated 24 h after cell seeding unless stated otherwise, and media were replaced every 48 h during prolonged assays to ensure consistent stimulus exposure.

### 2.11 Cell counting Kit-8 assay (CCK-8)

Cell viability was assessed using the CCK-8 (Yeasen, C1402240). Briefly, HCT-116 and HT-29 cells in logarithmic growth phase were seeded into 96-well plates at a density of 3 × 10^3^ cells per well (100 μL complete medium per well) and allowed to adhere for 24 h. Cells were then treated as described in Section 2.8 (Control, ISO, ISO + OMVs). Meanwhile, four parallel control interventions were simultaneously set up, including heat-inactivated *A. muciniphila* OMVs, blank medium processed with the same isolation protocol, OMV-depleted culture supernatant, and *E. coli*-derived OMVs at equal concentrations. Four parallel control samples were prepared for cell intervention experiments, with the same protein concentration (20 μg/mL) as native *A. muciniphila* OMVs, and all samples were filtered through a 0.22 μm membrane filter to eliminate residual bacterial contamination before use. (1) Heat-inactivated *A. muciniphila* OMVs: Purified *A. muciniphila* OMVs were inactivated by incubation at 95 °C for 30 min in a water bath to abolish their biological activity, then cooled to room temperature for subsequent use. (2) Blank medium processed with the same OMV isolation protocol: Blank CMC broth was subjected to the identical centrifugation, filtration and purification procedures as OMV isolation, without bacterial inoculation, to exclude the interference of medium components. (3) OMV-depleted culture supernatant: The cultured *A. muciniphila* bacterial suspension was first centrifuged at 8000×g for 10 min at 4 °C to remove bacterial pellets and large debris; the collected supernatant was then centrifuged at 12000×g for 30 min, followed by sequential filtration through 0.45 μm and 0.22 μm membrane filters to completely remove OMVs, and the final filtrate was used as OMV-depleted culture supernatant. (4) *E. coli*-derived OMVs: OMVs were isolated and purified from cultured *E. coli* using the same protocol as *A. muciniphila* OMVs, to exclude non-specific effects of OMVs from different bacterial species. Following incubation for 24, 48, or 72 h, 10 μL of CCK-8 solution was added to each well. Plates were gently shaken and returned to the incubator for 1.5 h in the dark. Absorbance at 450 nm was measured using a multi-mode microplate reader. All experiments were performed in biological triplicate, with each replicate including at least three technical replicates.

### 2.12 Colony formation assay

Cells (1,000/well) were seeded in 6-well plates and treated as above in Section 2.8 (Control, ISO, ISO + OMVs). Medium was refreshed every 3–4 days. After 10–14 days, colonies were fixed with 4% paraformaldehyde, stained with 0.1% crystal violet, and counted.

### 2.13 Transwell migration and invasion assay

For migration assays, serum-starved cells (2 × 10^4^ in 200 μL serum-free medium) were placed in the upper chamber of Transwell inserts (8.0 μm pores); and, the lower chamber contained 500 μL medium with 10% FBS. For invasion assays, inserts were pre-coated with Matrigel (BioCoat, 0321001). Cells were treated as indicated in Section 2.8 (Control, ISO, ISO + OMVs) and incubated for 48 h. Non-migrated/non-invaded cells were removed with a cotton swab. Cells on the lower surface were fixed, stained with crystal violet, and counted in four random fields per well.

### 2.14 Proteomic sample preparation

For subsequent proteomic analysis, cells were harvested following sequential washing steps. Briefly, the culture medium was aspirated, and the cells were washed three times with 10 mL of pre-chilled PBS to thoroughly remove residual serum proteins. After complete drainage, the cells were detached, suspended in cold PBS, and collected into a 15 mL conical tube. The suspension was centrifuged at 1000×g for 5–10 min at 4 °C. The resulting pellet was resuspended in 1 mL of fresh, cold PBS, transferred to a 1.5 mL microcentrifuge tube, and centrifuged again under the same conditions for 5 min. The final supernatant was completely aspirated, and the tight cell pellet was immediately flash-frozen in liquid nitrogen and shipped on dry ice, strictly avoiding repeated freeze–thaw cycles.

### 2.15 Enzyme-linked immunosorbent assay (ELISA)

The levels of Fetuin-A in the cell culture supernatants were quantified using a commercial ELISA kit according to the manufacturer’s instructions. Briefly, HCT-116 cells were treated with ISO for 48 h, after which the culture media from both the control and ISO groups were collected and centrifuged ( 1000×g , 10 min, 4 °C) to remove cellular debris. The resulting supernatants and standards were added to pre-coated microplates and incubated at room temperature. Following the washing steps, the detection antibody and HRP-conjugate were added. The colorimetric reaction was developed with TMB substrate and subsequently halted by the addition of stop solution. The optical density (OD) was measured at 450 nm using a microplate reader. The concentrations of Fetuin-A were calculated based on the standard curve and expressed as μg/mL.

### 2.16 OMV uptake assay

*A. muciniphila* OMVs were fluorescently labeled to visualize cellular internalization. Briefly, 1.5 mL of OMVs suspension (20 μg/mL total protein) was mixed with an equal volume of 5 μM DiI (1,1′-dioctadecyl-3,3,3′,3′-tetramethylindocarbocyanine perchlorate) solution and incubated for 1 h at room temperature in the dark. Unbound dye was removed by ultracentrifugation at 200000×g for 1.5 h at 4 °C. The labeled OMVs pellet was resuspended in pre-chilled PBS and stored protected from light until use.

For uptake experiments, human CRC cells (HCT-116 and HT-29) were seeded into confocal-compatible glass-bottom dishes at a density of 2 × 10^5^ cells per dish and allowed to adhere overnight. Then, they were treated with 100 μg/mL DiI-labeled OMVs ± 20 μM ISO. After 10 h of incubation, cells were gently washed three times with PBS to remove non-internalized vesicles and then fixed with 4% paraformaldehyde for 15 min at room temperature. Following fixation, cells were washed and stained for F-actin using ActinGreenTM 488 ReadyProbesTM Reagent (30 min, room temperature, protected from light). Nuclei were counterstained with DAPI (5 min). Finally, images were captured using a laser scanning confocal microscope to analyze the intracellular localization and uptake of the DiI-labeled *A. muciniphila* OMVs (red fluorescence).

### 2.17 Western blot

Cells or tissues were lysed in RIPA buffer (Beyotime) with protease inhibitors. Lysates were centrifuged ( 12000×g , 15 min, 4 °C), and 30 μg protein per sample was separated by 10% SDS-PAGE and transferred to PVDF membranes. Membranes were blocked and probed overnight at 4 °C with anti-Fetuin-A (Proteintech, 66,094-1-Ig, 1:2000) or anti-GAPDH (Proteintech, 60,004-1-Ig, 1:100,000), followed by HRP-conjugated secondary antibody (1:2500; ZB-2305). Bands were detected by ECL (Thermo Scientific, ZH399330A) and quantified using ImageJ.

### 2.18 Statistical analysis

All experimental data are presented as mean ± standard deviation (SD). Statistical analyses were performed using SPSS (version 27.0) and graphical representations were generated using GraphPad Prism (version 8.0). Data normality was assessed using the Shapiro–Wilk test. For normally distributed data, comparisons between two groups were conducted using an independent samples t-test, whereas comparisons among three or more groups were analyzed using two-way ANOVA. For non-normally distributed data, the nonparametric Mann – Whitney *U* test was applied. A *p*-value <0.05 was considered statistically significant.

## 3 Results

### 3.1 Chronic stress promotes CRC progression both *in vitro* and *in vivo*

**Figure 1:**
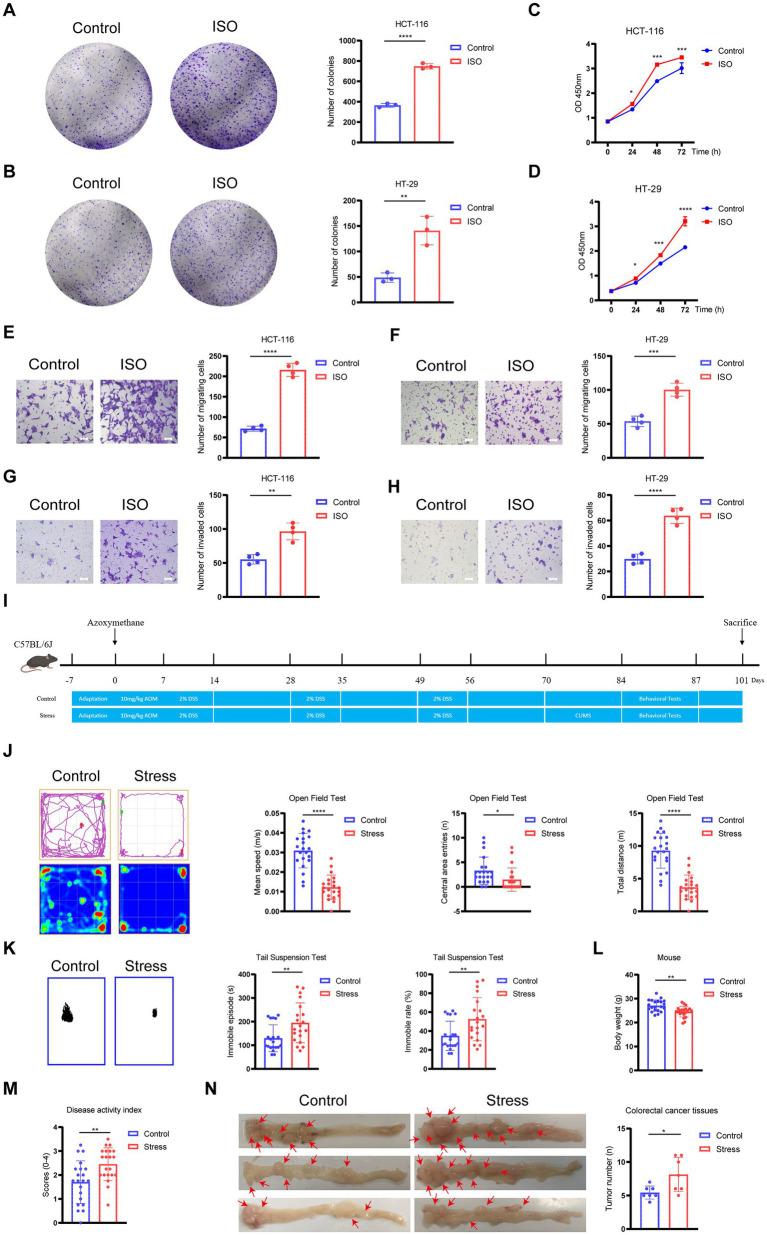
Chronic stress promotes colorectal cancer (CRC) progression both *in vitro* and *in vivo*. **(A,B)** Colony formation assay of HCT-116 **(A)** and HT-29 **(B)** cells treated with 20 μM isoproterenol (ISO) for 14 days. Representative images (left) and quantification of colony numbers (right) are shown. **(C,D)** Cell viability of HCT-116 **(C)** and HT-29 **(D)** cells treated with ISO for the indicated durations, measured by CCK-8 assay. **(E,F)** Migration of HCT-116 **(E)** and HT-29 **(F)** cells after ISO treatment for 48 h, evaluated by Transwell assay. Representative images (left) and quantification of migrated cells (right). **(G,H)** Invasion of HCT-116 **(G)** and HT-29 **(H)** cells after ISO treatment for 48 h, assessed using Matrigel-coated Transwell chambers. Representative images (left) and quantification of invaded cells (right). **(I)** Establishment of the chronic stress-induced colitis-associated CRC mouse model. **(J,K)** Anxiety- and depression-like behaviors in mice subjected to CUMS, evaluated by open-field test (OFT) **(J)** and tail-suspension test (TST) **(K)**. **(L)** Body-weight changes in experimental mice. **(M)** Disease activity index of experimental mice. **(N)** Gross morphology of colorectal tumors (left) and quantification of tumor numbers (right) in control and chronic-stress groups. Scale bar: 100 μm. Data are presented as mean ± SD. *p < 0.05, **p < 0.01, ***p < 0.001, ****p < 0.0001 vs. the ontrol group, as determined by Student’s *t*-test or two-way ANOVA.

To investigate the impact of chronic stress on CRC progression, we first simulated adrenergic signaling activation in vitro using ISO. Colony formation and CCK-8 assays demonstrated that ISO treatment significantly enhanced the proliferation of both HCT-116 (colony formation, *p* < 0.0001; CCK-8: 24 h: *p* < 0.05, 48 h: *p* < 0.001, 72 h: *p* < 0.001) and HT-29 cells (colony formation, *p* < 0.01; CCK-8: 24 h: *p* < 0.05, 48 h: *p* < 0.001, 72 h: *p* < 0.0001) compared with the control group (Figures 1A–D). Transwell assays confirmed that ISO increased the migratory and invasive capacities of HCT-116 (migration, *p* < 0.0001; invasion, *p* < 0.01) and HT-29 cells (migration, *p* < 0.001; invasion, *p* < 0.0001) (Figures 1E–H). These in vitro data indicate that chronic stress directly promotes key malignant phenotypes in CRC cells.

To validate these findings in vivo, we established a CRC mouse model subjected to CUMS (Figure 1I). Behavioral assessments revealed that stressed mice exhibited a significant reduction in total distance moved (*p* < 0.0001), average speed (*p* < 0.0001), and entries into the central zone (*p* < 0.05) in the OFT, along with prolonged immobility time in the TST (*p* < 0.01), confirming the successful induction of anxiety- and depression-like behaviors (Figures 1J,K). Physiologically, the stressed group exhibited significantly higher Disease Activity Index (DAI) scores and attenuated body-weight gain (both *p* < 0.01) (Figures 1L,M), indicating that chronic stress exacerbated intestinal inflammation and systemic health decline. Most importantly, mice exposed to chronic stress developed a significantly greater number of colorectal tumors than the control group (*p* < 0.05) (Figure 1N).

### 3.2 Chronic stress inhibits the uptake of *A. muciniphila* OMVs by CRC cells

**Figure 2:**
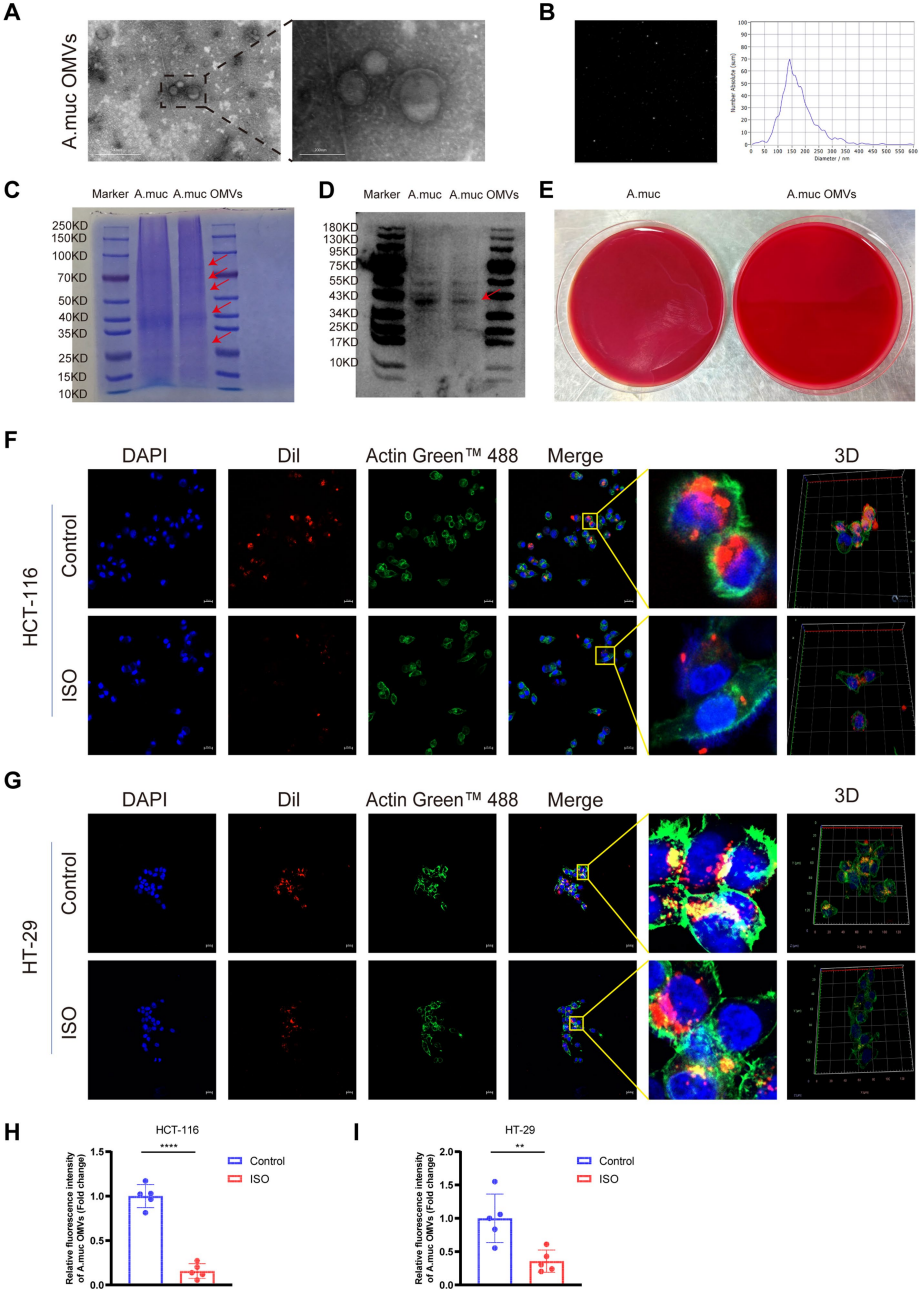
Chronic stress inhibits the uptake of *Akkermansia muciniphila* outer membrane vesicles (OMVs) by HCT-116 and HT-29 cells pretreated with or without 20 μM Isoproterenol (ISO) for 48 h. **(A,B)** Characterization of purified *A. muciniphila* OMVs via transmission electron microscopy and nanoparticle tracking analysis. **(C)** SDS-PAGE analysis of purified *A. muciniphila* OMVs to characterize their protein profile. **(D)** Western blotting analysis of purified *A. muciniphila* OMVs to detect the outer membrane marker LPS. **(E)** Sterility verification of purified OMVs; *A. muciniphila* culture (positive control) spread on anaerobic agar plates exhibited a dense bacterial lawn, while no bacterial growth was observed in the OMV group. **(F,G)** Representative fluorescence images of HCT-116 and HT-29 cells, including 3D reconstructions. Red: Dil-OMVs; green: F-actin (ActinGreen™ 488); blue: nuclei (DAPI). **(H)** Quantification of relative fluorescence intensity representing *A. muciniphila* OMVs uptake in HCT-116 cells (expressed as fold change relative to the control group) corresponding to panel **(F)**. **(I)** Quantification of relative fluorescence intensity representing *A. muciniphila* OMVs uptake in HT-29 cells (expressed as fold change relative to the control group) corresponding to panel **(G)**. Scale bars: 500 nm (**A** Left), 200 nm (**A** Right), and 20 μm **(F,G)**. Data are presented as mean ± SD. **p < 0.01, ****p < 0.0001 vs. the ontrol group, as determined by Student’s *t*-test.

Initially, TEM and NTA revealed that the extracted vesicles had an average diameter of 143.70 ± 85.2 nm and exhibited the characteristic cup-shaped and spherical bilayered nanostructures (Figures 2A,B). To further assess the biochemical composition of the OMVs, total proteins were separated by SDS-PAGE and stained with Coomassie brilliant blue. Compared with the whole-cell lysate of *A. muciniphila*, the OMV samples exhibited distinct protein enrichment patterns, with major protein bands concentrated in the range of 25–70 kDa, indicating that the OMVs carry a specific subset of bacterial proteins (Figure 2C). Western blot analysis was performed using anti-LPS antibody (Abmart, MK52671, 1:1000), and a specific target band was clearly detected at approximately 44 kDa, which further validated the successful isolation and purity of OMVs. (Figure 2D). Additionally, the sterility of the purified OMVs was verified by anaerobic cultivation. While the *A. muciniphila* culture (positive control) exhibited dense bacterial lawn growth on the anaerobic agar plates, no colonies were detected in the OMV group after 72 h of incubation, confirming that the OMV preparations were free of viable bacterial contamination (Figure 2E).

To determine whether stress signaling affects the interaction between CRC cells and bacterial vesicles, we examined the cellular internalization of DiI-labeled *A. muciniphila* OMVs using confocal microscopy. Compared with the control (OMVs alone), pretreatment with ISO significantly reduced the endocytosis of OMVs in both HCT-116 (*p* < 0.0001) and HT-29 (*p* < 0.01) cells (Figures 2F–I). This result suggests that chronic stress impairs the cellular uptake of *A. muciniphila* OMVs, potentially limiting their bioavailability under stress conditions.

### 3.3 Chronic stress promotes CRC progression by upregulating Fetuin-A expression

**Figure 3:**
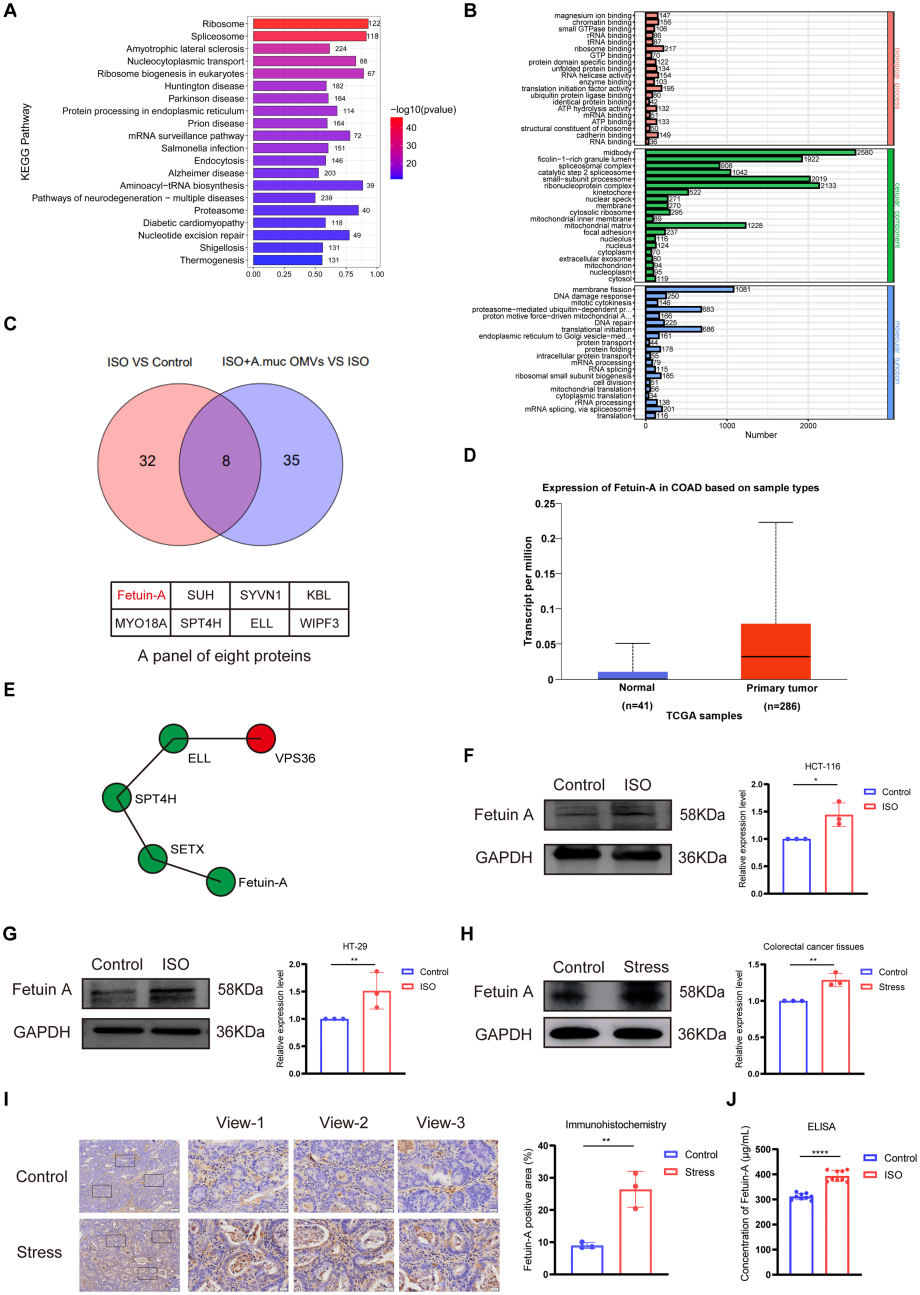
Chronic stress promotes colorectal cancer (CRC) progression by upregulating Fetuin-A expression. **(A,B)** KEGG and GO enrichment analysis of differentially expressed proteins in CRC cells in response to Isoproterenol (ISO) and *Akkermansia muciniphila* outer membrane vesicles (OMVs) treatment. **(C)** Venn diagram illustrating the intersection of differentially expressed proteins from proteomic analysis, identifying Fetuin-A as a key molecule commonly regulated by ISO and *A. muciniphila* OMVs. **(D)** TCGA samples consist of clinically collected primary tumor tissues and matched adjacent normal tissues from cancer patients. **(E)** Protein–protein interaction network of Fetuin-A and its interacting proteins, revealing two regulatory cascades including SPT4H-ELL-VPS36 for vesicle trafficking and SPT4H-SETX-Fetuin-A for transcriptional modulation of Fetuin-A. **(F,G)** Western blot analysis and quantification of Fetuin-A protein levels in HCT-116 **(F)** and HT-29 **(G)** cells treated with 20 μM ISO for 48 h. **(H)** Western blot analysis and quantification of Fetuin-A expression in tumor tissues from control and stressed mice. GAPDH served as the loading control for western blots. **(I)** Immunohistochemical staining of Fetuin-A in CRC tissues from control and stressed mice. **(J)** Enzyme-linked immunosorbent assay of Fetuin-A in HCT-116 supernatants treated with or without ISO. Scale bar: 100 μm. Data are mean ± SD. *p < 0.05, **p < 0.01, ****p < 0.0001 vs. the ontrol group, as determined by Student’s *t*-test.

To identify key proteins mediating the observed effects, we performed a comparative proteomic analysis. Kyoto Encyclopedia of Genes and Genomes (KEGG) pathway enrichment analysis revealed that *A. muciniphila* OMVs and ISO predominantly modulate pathways linked to neurodegenerative disorders and the biosynthetic processing of cellular proteins and RNA (Figure 3A). Gene Ontology (GO) analysis revealed that the differentially expressed genes were predominantly enriched in molecular functions (e.g., structural constituent of ribosome, ATP hydrolysis), cellular components (e.g., spliceosome, ribonucleoprotein complex), and biological processes (e.g., mRNA splicing, DNA damage response, and protein ubiquitination) (Figure 3B). These results suggest that OMVs and ISO significantly modulate core biological processes, including gene expression, protein synthesis and metabolism, as well as cellular stress repair. By intersecting the differentially expressed proteins induced by ISO with those modulated by OMVs under ISO stimulation, we identified eight candidate molecules commonly regulated by both stimuli (Figure 3C). Transcriptional expression of Fetuin-A, quantified as Transcripts Per Million, was evaluated using the UALCAN database. Analysis of 286 primary colon adenocarcinoma (COAD) samples and 41 normal controls revealed a significant upregulation of Fetuin-A in tumor tissues compared to their normal counterparts (Figure 3D). To explore the potential molecular mechanism underlying OMV-mediated regulation of Fetuin-A, we constructed the protein–protein interaction (PPI) network of Fetuin-A and correlated proteins (Figure 3E). The network revealed two distinct regulatory cascades associated with Fetuin-A. First, the transcriptional regulators SPT4H and ELL interacted with VPS36, forming a transcriptional-vesicle regulatory cascade. VPS36 acts as a core component of the ESCRT complex and participates in endosomal sorting and vesicle trafficking, suggesting that OMV exposure may modulate host vesicle transport machinery through transcriptional regulatory factors. Second, the RNA-processing factor SETX exhibited direct interaction with Fetuin-A, forming another regulatory pathway involved in transcriptional modulation. Collectively, these PPI analyses indicated that transcriptional regulation and vesicle trafficking pathways constitute two core downstream regulatory modules. OMVs may interfere with transcriptional elongation-related proteins, thereby regulating vesicle transport and inflammatory signaling, and ultimately modulating Fetuin-A expression and cellular responses. Based on its established association with CRC progression and the availability of recombinant protein for functional validation, Fetuin-A was selected for further investigation.

Western blot analysis confirmed that ISO treatment significantly upregulated Fetuin-A protein levels in both HCT-116 (*p* < 0.05) and HT-29 (*p* < 0.01) cells (Figures 3F,G). *In vivo*, tumor tissues from the chronic-stress group also exhibited significantly higher Fetuin-A expression compared with the control group (*p* < 0.01) (Figure 3H). Immunohistochemical staining further corroborated these findings, showing stronger and more extensive positive staining for Fetuin-A in tumors from stressed mice (*p* < 0.01) (Figure 3I). ELISA further corroborated these findings, revealing significantly elevated concentrations of Fetuin-A in the supernatants of HCT-116 cells following ISO treatment (*p* < 0.0001) (Figure 3J). Together, these data indicate that chronic stress promotes CRC progression, at least in part, through upregulation of Fetuin-A.

### 3.4 *A. muciniphila* OMVs antagonize chronic stress-induced CRC progression *in vitro* and *in vivo*

**Figure 4:**
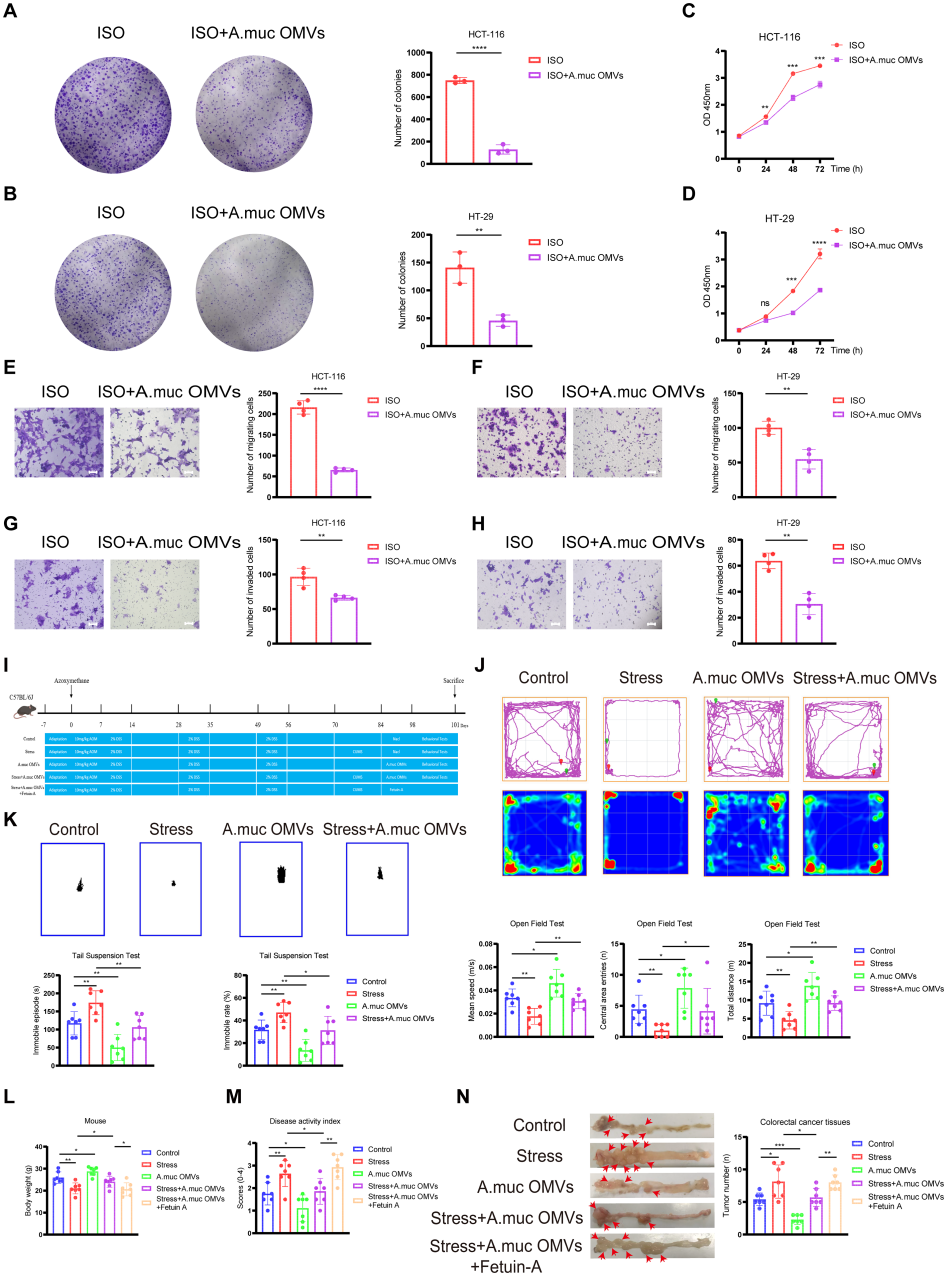
*Akkermansia muciniphila* outer membrane vesicles (OMVs) antagonize chronic stress-induced colorectal cancer (CRC) progression *in vitro* and *in vivo*. **(A,B)** Colony formation assay of HCT-116 **(A)** and HT-29 **(B)** cells treated with Isoproterenol (ISO) (20 μM) alone or in combination with *A. muciniphila* OMVs (20 μg/mL). **(C,D)** CCK-8 assay assessing proliferation of HCT-116 **(C)** and HT-29 **(D)** cells under the indicated treatments. **(E, F)** Transwell migration assay of HCT-116 I and HT-29 **(F)** cells treated as above. **(G,H)** Matrigel-coated Transwell invasion assay of HCT-116 **(G)** and HT-29 **(H)** cells. **(I)** Schematic diagram of *A. muciniphila* OMV gavage and Fetuin-A tail vein injection. **(J,K)** Anxiety- and depression-like behaviors in mice subjected to CUMS with or without *A. muciniphila* OMV treatment, evaluated by open-field test (OFT) **(J)** and tail-suspension test (TST) **(K)**. **(L)** Body-weight changes in experimental mice. **(M)** Disease activity index of experimental mice. **(N)** *In vivo* therapeutic efficacy of *A. muciniphila* OMVs in the AOM/DSS-induced CRC mice with or without CUMS. Representative colon images (left) and quantification of tumor numbers (right) are shown for each treatment group: Control, Stress, OMVs, Stress + OMVs, and Stress + OMVs + exogenous Fetuin-A. Scale bar: 100 μm. Data are presented as mean ± SD. ^ns^ *p* > 0.05, *p < 0.05, **p < 0.01, ***p < 0.001, ****p < 0.0001. Statistical significance was determined by Student’s *t*-test or two-way ANOVA.

We next examined whether *A. muciniphila* OMVs could counteract the pro-tumorigenic effects of chronic stress. *In vitro*, colony formation and CCK-8 assays demonstrated that co-treatment with OMVs significantly suppressed the ISO-induced proliferation of both HCT-116 (colony formation, *p* < 0.0001; CCK-8: 24 h: *p* < 0.01, 48 h: *p* < 0.001, 72 h: *p* < 0.001) and HT-29 cells (colony formation, *p* < 0.01; CCK-8: 24 h: *p* > 0.05, 48 h: *p* < 0.001, 72 h: *p* < 0.0001) compared with the ISO-treated group (Figures 4A–D). Transwell assays confirmed that OMVs effectively attenuated the ISO-enhanced migratory and invasive capacities of HCT-116 (migration, *p* < 0.0001; invasion, *p* < 0.01) and HT-29 cells (migration, *p* < 0.01; invasion, *p* < 0.01) (Figures 4E–H). No significant inhibition of proliferation (Supplementary Figures 1A,B), migration (Supplementary Figures 1C,D), or invasion (Supplementary Figures 1E,F) was observed in ISO-induced cancer cells treated with heat-inactivated *A. muciniphila* OMVs, blank medium processed with the same isolation protocol, OMV-depleted culture supernatant, or *E. coli*-derived OMVs. These findings confirm that the effects are specifically induced by biologically active *A. muciniphila* OMVs. These in vitro data indicated that *A. muciniphila* OMVs effectively counteracted the ISO-mediated promotion of malignant phenotypes in CRC cells.

*In vivo*, chronic stress-induced CRC mice were treated with *A. muciniphila* OMVs (via oral gavage for 14 days), either alone or in combination with Fetuin-A (via tail vein injection) (Figure 4I). Behavioral assessments showed that CUMS induced marked anxiety- and depression-like phenotypes, characterized by reduced locomotor activity and central exploration in the OFT (*p* < 0.01), and increased despair-like behavior in the TST (*p* < 0.01). Interestingly, *A. muciniphila* OMVs administration alone effectively enhanced exploratory activity and reduced immobility (*p* < 0.05 or *p* < 0.01). More importantly, OMV treatment significantly ameliorated stress-induced impairments, leading to substantial improvements in OFT parameters (total distance and speed, *p* < 0.01; central entries, *p* < 0.05) and a dramatic decrease in TST immobility (*p* < 0.05). These findings suggest that *A. muciniphila* OMVs exert potent anxiolytic- and antidepressant-like effects (Figures 4J,K). Physiologically, the stressed group exhibited significantly higher DAI scores (*p* < 0.01) and marked body-weight loss (*p* < 0.01). Conversely, administration of *A. muciniphila* OMVs to these stressed mice effectively reversed the weight loss (*p* < 0.05) and lowered the DAI scores (*p* < 0.05), indicating that *A. muciniphila* OMV treatment significantly mitigated the pathological severity. Notably, the further administration of exogenous Fetuin-A to this group significantly reversed these protective effects, leading to increased weight loss (*p* < 0.05) and elevated DAI scores (*p* < 0.01), which suggests that Fetuin-A acts as a negative regulator of OMV-mediated protection (Figures 4L,M). Most importantly, chronic stress significantly increased tumor number (*p* < 0.05), whereas administration of *A. muciniphila* OMVs alone markedly suppressed tumor formation (*p* < 0.001). Notably, co-administration of OMVs in stressed mice reversed the stress-induced increase in tumor burden (*p* < 0.05). However, the addition of exogenous Fetuin-A to this group led to a significant increase in tumor numbers (*p* < 0.01), indicating that Fetuin-A effectively negates the anti-tumor effects of *A. muciniphila* OMVs (Figure 4N). These results demonstrate that *A. muciniphila* OMVs functionally antagonize chronic stress-induced CRC progression, whereas Fetuin-A acts as a pro-tumorigenic factor that promotes the advancement of the disease.

### 3.5 *A. muciniphila* OMVs counteract chronic stress-induced CRC progression by downregulating Fetuin-A expression

**Figure 5:**
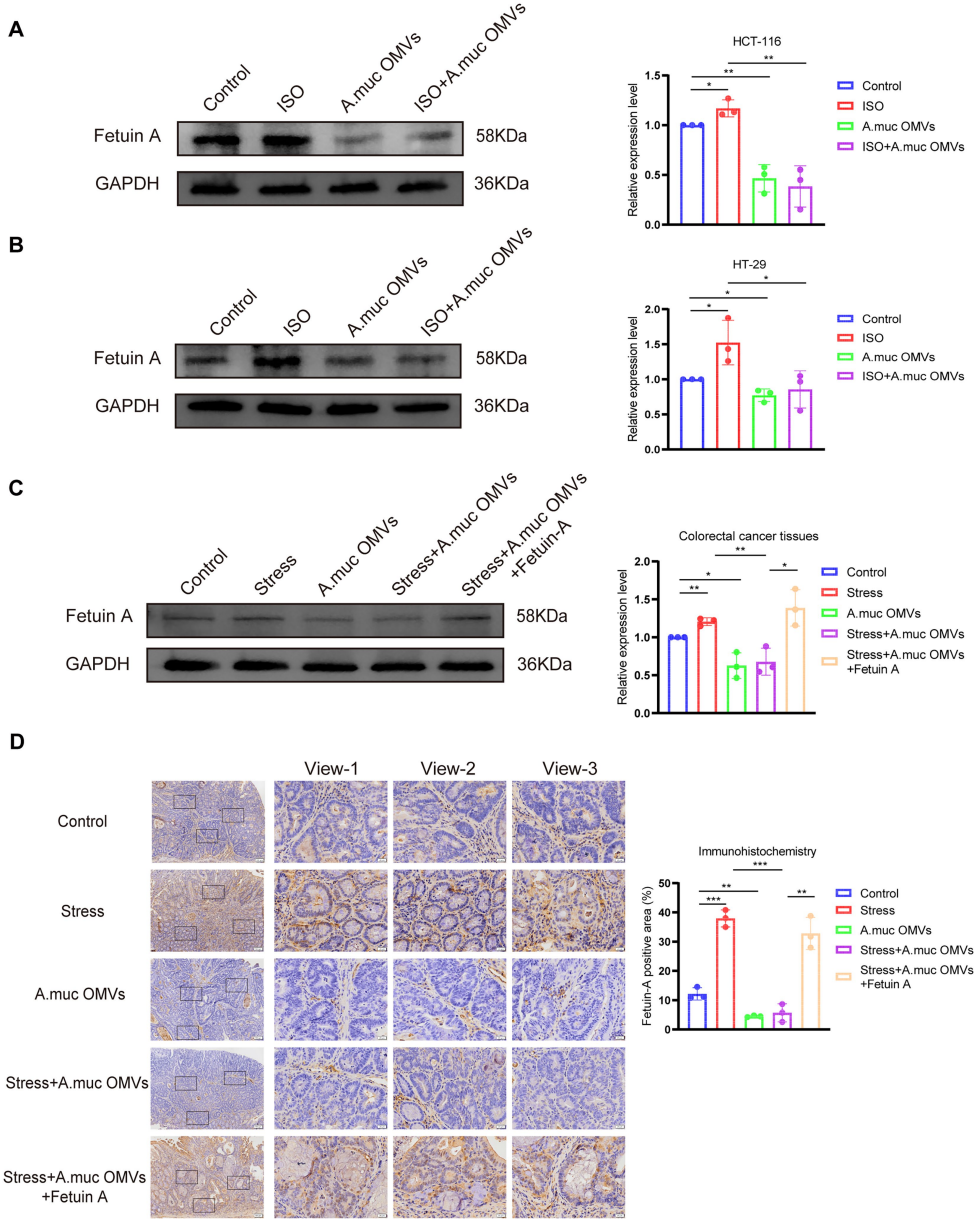
*Akkermansia muciniphila* outer membrane vesicles (OMVs) counteract chronic stress-induced colorectal cancer (CRC) progression by downregulating Fetuin-A expression. **(A,B)** Western blot analysis and quantification of Fetuin-A protein levels in HCT-116 **(A)** and HT-29 **(B)** cells under different treatments: control, isoproterenol (ISO) (20 μM), OMVs (20 μg/mL), and ISO + OMVs. **(C)** Western blot analysis and quantification of Fetuin-A expression in tumor tissues from the following *in vivo* groups: control, stress, OMVs, stress + OMVs, and stress + OMVs + exogenous Fetuin-A. GAPDH was used as the loading control. **(D)** Immunohistochemical staining of Fetuin-A in CRC tissues from the corresponding *in vivo* groups. Scale bar: 100 μm. Data are presented as mean ± SD. *p < 0.05, **p < 0.01, ***p < 0.001. Statistical significance was determined by two-way ANOVA.

To elucidate the molecular mechanisms underlying the therapeutic effect of *A. muciniphila* OMVs, we focused on Fetuin-A regulation. *In vitro*, Western blot analysis showed that ISO-induced upregulation of Fetuin-A in HCT-116 (*p* < 0.05) and HT-29 (*p* < 0.05) cells was effectively reversed by co-treatment with *A. muciniphila* OMVs (HCT-116: *p* < 0.01; HT-29: *p* < 0.05 vs. ISO group). Moreover, OMVs alone significantly reduced basal Fetuin-A expression (HCT-116: *p* < 0.01; HT-29: *p* < 0.05 vs. Control group) (Figures 5A,5B).

*In vivo*, Fetuin-A protein levels were significantly elevated in tumor tissues from the chronic-stress group (*p* < 0.01). In contrast, OMV intervention significantly decreased its expression (*p* < 0.05). Critically, in stressed mice, administration of OMVs suppressed the stress-induced upregulation of Fetuin-A (*p* < 0.01 vs. Stress group). Notably, the further administration of exogenous Fetuin-A to this group significantly re-elevated its expression levels (*p* < 0.05 vs. Stress + OMVs group) (Figure 5C). Immunohistochemistry results were consistent, showing strong Fetuin-A positivity in the stress and exogenous Fetuin-A groups, which was markedly reduced by OMV treatment in stressed animals (Figure 5D).

Collectively, these findings indicate that chronic stress promotes CRC progression via upregulation of Fetuin-A, and that *A. muciniphila* OMVs exert their anti-tumor effects by downregulating this key pro-tumorigenic protein.

## 4 Discussion

**Figure 6:**
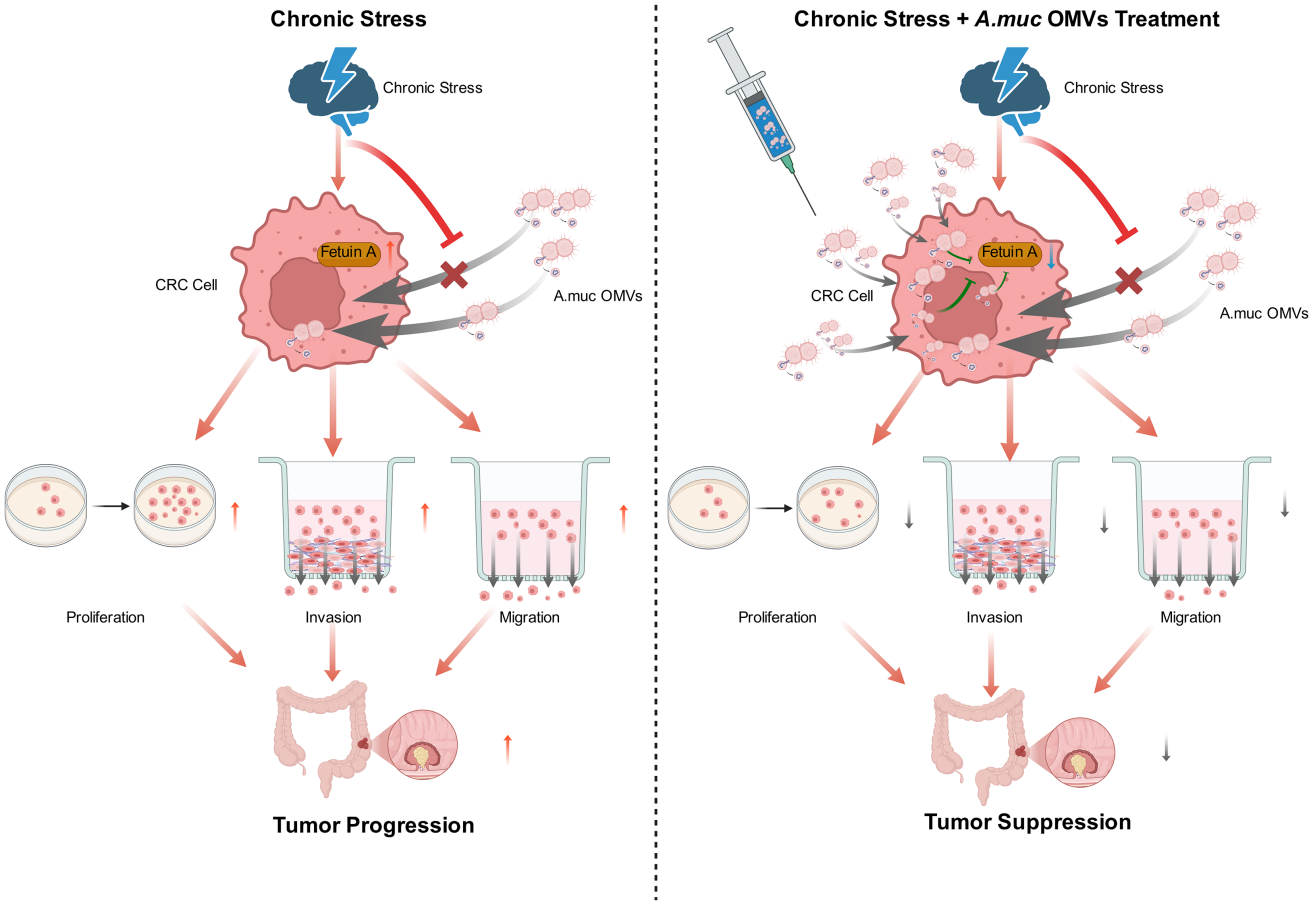
Proposed mechanistic model of the “stress–Fetuin-A–colorectal cancer (CRC)” axis and its intervention by *Akkermansia muciniphila* outer membrane vesicles (OMVs). (Left) Chronic stress upregulates the expression of the pro-tumorigenic protein Fetuin-A in CRC cells. Elevated Fetuin-A promotes key malignant phenotypes, including enhanced proliferation, invasion, and migration, thereby accelerating tumor progression. (Right) Administration of *A. muciniphila* OMVs counteracts this pathway. The OMVs are internalized by CRC cells and downregulate Fetuin-A expression. This reduction in Fetuin-A levels reverses the pro-malignant effects induced by chronic stress, ultimately leading to suppressed tumor growth. This model summarizes the central finding that *A. muciniphila* OMVs ameliorate stress-exacerbated CRC progression by targeting Fetuin-A.

This study provides the first evidence that OMVs derived from the probiotic bacterium *A. muciniphila* inhibit chronic stress-exacerbated CRC progression by downregulating the pro-tumorigenic protein Fetuin-A (Figure 6). Our findings delineate a novel “stress–Fetuin-A–CRC” regulatory axis and position *A. muciniphila* OMVs as a promising therapeutic strategy for managing stress-associated malignancies.

At the cellular level, we confirmed that the *β*-adrenergic agonist ISO significantly promoted the proliferation, migration, and invasion of CRC cells, which aligns with established roles of adrenergic signaling in tumor progression (Guan et al., 2023; Cao et al., 2021; Ghosn et al., 2025; Zhou et al., 2022). Notably, we made the novel observation that ISO pretreatment markedly impaired the cellular uptake of *A. muciniphila* OMVs. This finding suggests that the stress-shaped tumor microenvironment may not only directly drive malignancy but also compromise the bioavailability and efficacy of beneficial microbial derivatives, potentially by altering vesicle-cell interaction dynamics.

The biogenesis, release, and cellular uptake of bacterial OMVs are dynamic processes regulated by a complex interplay of environmental cues and intrinsic bacterial factors (Zhang X. et al., 2025). Environmental stressors primarily drive OMV production through several mechanisms: direct physical perturbation of membrane architecture, induction of cellular stress responses, and alteration of cellular metabolism that affects membrane component synthesis. For instance, sub-lethal antibiotic exposure can stimulate OMV release as a bacterial defense mechanism (Tian et al., 2023). Endogenous regulation is equally critical, as exemplified by small RNAs inhibiting outer membrane protein translation to promote vesiculation in *Vibrio cholerae* (Sorgenfrei et al., 2025; Zingl et al., 2021; Xiu et al., 2025), and quorum-sensing molecules coordinating vesicle release for collective behaviors (Zhao Z. et al., 2022; Tu et al., 2025). Conversely, OMV release can be suppressed by high-intensity stressors that reduce viable cell numbers (Lin et al., 2025; Salamaga et al., 2021), by physiological states like biofilm formation that physically constrain vesicle dissemination (Chen N. et al., 2024; Ñahui Palomino et al., 2021), or by conditions that restore membrane stability and cellular homeostasis (Winkle et al., 2021; Wang et al., 2024; Huete-Acevedo et al., 2024).

The efficacy of OMV-mediated communication further depends on recipient cell uptake, a process regulated by vesicle properties, microenvironmental conditions, and recipient cell state. OMVs with poor mechanical stability may be cleared before reaching target cells (Caselli et al., 2021; Sun et al., 2024; Fu et al., 2021), while their cargo composition directly influences uptake efficiency (Mas-Bargues and Borrás, 2021; Xie et al., 2024). The local microenvironment, such as the acidic pH of the tumor microenvironment, can enhance vesicle retention and internalization (Gong et al., 2021). Additionally, the functional state of recipient cells—for example, macrophage polarization—determines their capacity for OMV uptake (Fan et al., 2024; Zhang Y. et al., 2025). Taken together, this regulatory framework suggests that chronic stress may impair the uptake of *A. muciniphila* OMVs by CRC cells through multifaceted effects, potentially altering the bacterial biofilm, the physicochemical properties and cargo of OMVs, and the physiological state of the recipient cancer cells.

As the central regulatory molecule identified in this study, Fetuin-A exhibits significant pro-tumorigenic properties. Fetuin-A is a multifunctional glycoprotein synthesized primarily by the liver, and its role as a pro-oncogenic factor has been documented in various malignancies (Zhang P. et al., 2025; Hsu et al., 2024; Kan et al., 2022). In the context of CRC, its expression is significantly upregulated in tumor tissues and strongly correlates with advanced clinical stages and poor prognosis (Winter et al., 2021). Mechanistically, Fetuin-A is known to drive malignant progression by modulating diverse pathways, including cell cycle regulation, apoptosis inhibition, and extracellular matrix remodeling (Gerst et al., 2021). The innovative aspect of our research lies in being the first to connect Fetuin-A with chronic stress signaling and the interventional effects of *A. muciniphila* OMVs, which are natural nano-sized vesicles rich in diverse bioactive molecules (Liu H. et al., 2022; Xiao et al., 2024). We found that while ISO induced the upregulation of Fetuin-A in CRC cells, co-treatment with *A. muciniphila* OMVs effectively reversed this effect. This result clearly indicates that Fetuin-A serves as a key molecular target through which *A. muciniphila* OMVs antagonize the pro-cancer effects of chronic stress. It can be inferred that *A. muciniphila* OMVs may suppress downstream pro-proliferative and pro-invasive pathways activated by stress signals through the downregulation of Fetuin-A. However, the precise mechanism by which *A. muciniphila* OMVs regulate Fetuin-A expression warrants further investigation. Potential mechanisms may involve specific microRNAs or proteins carried by the OMVs that target Fetuin-A mRNA or influence its transcriptional regulation. Based on the PPI analysis, the present study revealed two key regulatory cascades: the SPT4H-ELL-VPS36 pathway associated with vesicle trafficking and endosomal sorting, and the SPT4H-SETX-Fetuin-A cascade involved in transcriptional modulation. Each factor exerts unique biological functions. ELL participates in transcriptional regulation by recruiting AF4, EAF1 and p53 via its C-terminal domain (Takahashi et al., 2021). The conserved elongation factor Spt4H interacts with Spt5 to modulate RNA polymerase II elongation, nucleosome traversal, and chromatin stability (Uzun et al., 2021). As a core ESCRT component, VPS36 mediates endosomal trafficking and cargo sorting (Lin et al., 2023). Arl4A binds VPS36 to sustain ESCRT-III assembly, thereby facilitating EGFR degradation (Lin et al., 2023). SETX acts as an ATP-dependent helicase to resolve pathological R-loops and prevent their abnormal accumulation (Hasanova et al., 2023; Gatti et al., 2023). These findings suggest that *A. muciniphila* OMVs may interfere with host transcriptional elongation and vesicle transport processes, thereby modulating Fetuin-A expression and influencing downstream inflammatory and cellular functional responses. Building on these preliminary results, further mechanistic investigations can be designed in future studies. Comprehensive component analysis of OMVs will be performed, including quantitative proteomic profiling and small RNA (miRNA) sequencing. This approach aims to systematically identify the functional cargoes (proteins and non-coding RNAs) carried by OMVs and elucidate their potential roles in mediating host cell responses. Combined proteomic analysis can be utilized to systematically screen OMV-responsive downstream molecules and clarify the regulatory network of vesicle trafficking related to Fetuin-A. In addition, targeted genetic intervention can be applied to verify the functional role of Fetuin-A in colorectal cancer cells. Specifically, knockdown of Fetuin-A expression in colorectal cancer cell lines can be performed to investigate the essential role of Fetuin-A in mediating OMV-induced cellular changes, including tumor cell proliferation, migration and inflammatory responses. Combined with interference of key vesicle trafficking factors, such as VPS36, subsequent studies can further clarify the causal relationship between OMVs, vesicle transport pathways and Fetuin-A-mediated biological functions. Such validation experiments will provide more robust evidence to clarify the molecular mechanism underlying *A. muciniphila* OMVs-mediated regulation of Fetuin-A in colorectal cancer.

Our *in vitro* findings were robustly validated by *in vivo* animal experiments. In a mouse model of CUMS, we observed that chronic stress significantly accelerated CRC tumor growth, a result consistent with clinical observations (Guan et al., 2023; Cao et al., 2024). Importantly, systemic administration of *A. muciniphila* OMVs not only effectively inhibited stress-induced tumor growth but also significantly reduced Fetuin-A protein levels in tumor tissues. This high degree of consistency between in vitro and in vivo results strongly supports the validity of the “*A. muciniphila* OMVs – Fetuin-A” regulatory axis under pathophysiological conditions and highlights the considerable potential of *A. muciniphila* OMVs as a novel therapeutic strategy. Compared to conventional chemotherapeutic agents, OMVs derived from commensal bacteria offer advantages in biocompatibility and low toxicity (Meng et al., 2024; Gilmore et al., 2022). Their mechanism of action, which involves precise targeting of Fetuin-A, paves a new avenue for developing personalized, low-side-effect treatment regimens for CRC patients experiencing psychological stress.

Despite the significant findings, this study has several limitations that should be addressed in future research. First, our work primarily utilized established CRC cell lines; validation with patient-derived primary tumor cells or organoid models would enhance the clinical translational relevance of the conclusions. Second, while we identified Fetuin-A as a key node, the specific downstream signaling pathways (e.g., PI3K/AKT or ERK1/2) affected by its OMV-mediated downregulation (Hsu et al., 2024) remain to be fully elucidated and require further investigation through experiments such as co-immunoprecipitation and kinase activity assays. Finally, the key active components within *A. muciniphila* OMVs responsible for the observed regulatory effect are still uncharacterized. Identifying and validating these functional molecules through future multi-omics analyses will be a critical step toward developing them into defined therapeutics.

## 5 Conclusion

This study revealed for the first time that OMVs derived from *A. muciniphila* effectively ameliorate chronic stress-exacerbated CRC progression by specifically downregulating the pro-tumorigenic protein Fetuin-A. Through integrated in vitro and in vivo approaches, we established a pivotal “*A. muciniphila* OMVs → Fetuin-A” regulatory axis. Our findings demonstrate that chronic stress not only promotes CRC malignancy but also impairs the cellular uptake of OMVs. Importantly, *A. muciniphila* OMVs counteract these effects by reversing stress-induced Fetuin-A overexpression, thereby suppressing tumorigenesis. Collectively, this work systematically elucidates a novel mechanism through which probiotic-derived OMVs antagonize stress-induced tumor progression. The discovery provides a crucial molecular link within the intricate “gut microbiota–host stress–tumor” network and offers a solid experimental foundation for developing *A. muciniphila* OMVs as a novel biotherapeutic strategy for stress-induced CRC.

## Data Availability

The raw data supporting the conclusions of this article will be made available by the authors, without undue reservation.

## Supplementary material

SUPPLEMENTARY FIGURE 1 Control treatments fail to antagonize ISO-induced proliferation, migration, and invasion in colorectal cancer (CRC) cells. **(A,B)** CCK‑8 assay assessing proliferation of HCT‑116 **(A)** and HT‑29 **(B)** cells treated with Isoproterenol (ISO) (20 μM) alone or in combination with various control samples. **(C,D)** Transwell migration assay of HCT‑116 **(C)** and HT‑29 **(D)** cells under the indicated treatments. **(E,F)** Matrigel‑coated Transwell invasion assay of HCT‑116 **(E)** and HT‑29 **(F)** cells. Control treatments include heat-inactivated *A. muciniphila* OMVs, blank medium subjected to the same OMV isolation procedure, OMV-depleted culture supernatant, and *E. coli*-derived OMVs, all administered at a consistent concentration of 20 μg/mL. Data are presented as mean ± SD. ^ns^*p* > 0.05. Statistical significance was determined by Student’s *t*-test or one-way ANOVA.

SUPPLEMENTARY DATA SHEET S1 16S rRNA gene sequence of *Akkermansia muciniphila* ATCC BAA-835.
