# Supplementary figures and images for: Outer membrane vesicles from Akkermansia muciniphila antagonize chronic stress-induced colorectal cancer progression by downregulating Fetuin-A
Source: Front Microbiol. 2026 May 22;17:1821362. doi: 10.3389/fmicb.2026.1821362 (PMC13236662; doi:10.3389/fmicb.2026.1821362)

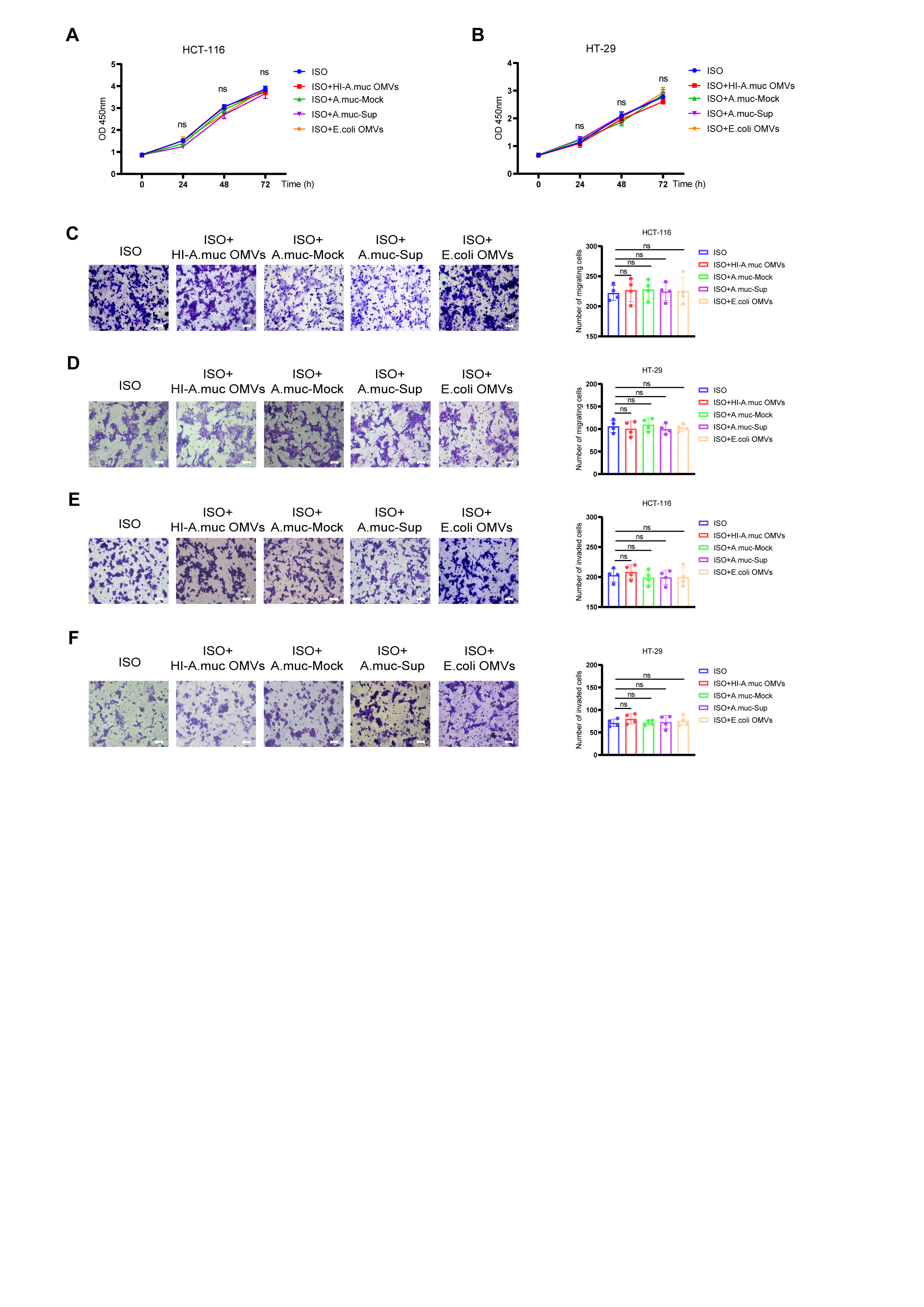

Supplement: SUPPLEMENTARY FIGURE 1 — Control treatments fail to antagonize ISO-induced proliferation, migration, and invasion in colorectal cancer (CRC) cells. (A,B) CCK‑8 assay assessing proliferation of HCT‑116 (A) and HT‑29 (B) cells treated with Isoproterenol (ISO) (20 μM) alone or in combination with various control samples. (C,D) Transwell migration assay of HCT‑116 (C) and HT‑29 (D) cells under the indicated treatments. (E,F) Matrigel‑coated Transwell invasion assay of HCT‑116 (E) and HT‑29 (F) cells. Control treatments include heat-inactivated A. muciniphila OMVs, blank medium subjected to the same OMV isolation procedure, OMV-depleted culture supernatant, and E. coli-derived OMVs, all administered at a consistent concentration of 20 μg/mL. Data are presented as mean ± SD. nsp > 0.05. Statistical significance was determined by Student’s t-test or one-way ANOVA. [file Image_1.TIF]
