# Supplementary material for: Outer membrane vesicles from Akkermansia muciniphila antagonize chronic stress-induced colorectal cancer progression by downregulating Fetuin-A
Source: Front Microbiol. 2026 May 22;17:1821362. doi: 10.3389/fmicb.2026.1821362 (PMC13236662; doi:10.3389/fmicb.2026.1821362)
Supplement: SUPPLEMENTARY DATA SHEET S1 — 16S rRNA gene sequence of Akkermansia muciniphila ATCC BAA-835. [file Data_Sheet_1.DOCX]

16S rRNA gene sequence of *Akkermansia muciniphila* ATCC BAA-835

TTAGGACCCTGCCTCCTTGCGGTTGGCTTCAGATACTTCGGGTGCGACCGGCTTCCATGATGTGACGGGCGGTGTGTACAAGACCCGGGAACGTATTCACGGCGCCGTAGCTGATGCGCCATTACTAGCGATTCCGGCTTCGTGTAGGCGGGTTGCAGCCTACAGTCCGAACTGGGCCCAGTTTTTAGGATTTCCTCCGCCTCGCGGCTTCGGCCCCCTCTGTACTGGGCATTGTAGTACGTGTGCAGCCCTGGGCATAAGGGCCATACTGACCTGACGTCGTCCCCACCTTCCTCCCAGTTGATCTGGGCAGTCTCGCCAGAGTCCCCACCTTCACGTGCTGGTAACTGGCAACAGGGGTTGCGCTCGTTGCTGGACTTAACCAAACATCTCACGACACGAGCTGACGACGGCCATGCAGCACCTGTGTAACGCCTCCGAAGAGTCGCATGCTTTCACATGTTGTTCATTACATGTCAAGCCCAGGTAAGGTTCTTCGCGTTGCATCGAATTAAGCCACATACTCCACCGCTTGTGCGGGTCCCCGTCAATTTCTTTGAGTTTTAATCTTGCGACCGTACTCCCCAGGCGGCACGCTTAACGCGTTAGCTCCGGCACGCAGGGGGTCGATTCCCCGCACACCAAGCGTGCACCGTTTACTGCCAGGACTACAGGGGTATCTAATCCCTTTCGCTCCCCTGGCCTTCGTGCCTCAGCGTCAGTTAATGTCCAGGAACCCGCCTTCGCCACGAGTGTTCCTCTCGATATCTACGCATTTCACTGCTACACCGAGAATTCCGGTTCCCCCTCCATTACTCTAGTCTCGCAGTATCATGTGCCGTCCGCGGGTTGAGCCCGCGCCTTTCACACACGACTTACGAAACAGCCTACGCACGCTTTACGCCCAGTGATTCCGAACAACGCTTGAGACCTCTGTATTACCGCGGCTGCTGGCACAGAGTTAGCCGTCTCTTCCTCTTGTGGTACTATCTTTTTAATTTGCTCCCACATGACAGGGGTTTACAATCCGAAGACCTTCATTCCCCCACGCGGCGTCGCACCATCAGGGTTTCCCCCATTGTGAATGATTCTCGACTGCTGCCACCCGTAGGTGTCTGGACCGTGTCTCAGTTCCAGTGTGGCCGGACATCCTCTCAGACCGGCTACCCGTCATCGCCTTGGTGAGCCGTTACCTCACCAACTAACTAATAGGCCGCGAGCCCATCCCCAAGCGCATTGCTGCTTTAATCTTTCGATACTATGCGGTATTAATCCCAGTTTCCCAGGGCTATCCCGCTCTCGGGGGCAGGTTACTCACGTGTTACTCACCCGTGCGCCACTAGAGAATTATTAGCAAGCTAGCAATTCTCTCGTTCGACTTGCA
